# The Position of Employed Contributors Now Entering Insurance

**Published:** 1914-02-21

**Authors:** 


### NATIONAL INSURANCE ACT DEVELOPMENTS.

### The Position of Employed Contributors Now Entering Insurance.

We are reminded by a circular recently issued by the Insurance Commissioners (A.S. 122) that one of the advantages conferred by the Amendment Act of 1913 upon those entitled to become employed contributors has now ceased to operate. We refer to the extension of the time for entry into insurance at full rates of benefit for persons over seventeen years of age that was granted by Section 2 of the Act of 1913.

It will be remembered that under the Act of 1911 the right of entry into insurance at full rates of benefit for the level contribution at all ages at entry extended for twelve months only after the Act came into operation. This term would have expired in July last, but by the Amendment Act the concession for entry on these terms was extended to October 12 last.

The number of employed contributors insured under the provisions of the Acts is constantly increasing, for as young persons who are in employment attain the age of sixteen they automatically come within the operation of the Acts, and contributions have to be paid by them and on their behalf in the same manner as for those who became insured when the principal Act first came into force. So far as these young persons are concerned there is no reduction from the full rate of benefit, as conferred by the Act, on account of the deferment of their entry into insurance. In point of fact the benefits and contributions are related to each other on the supposition that all persons enter insurance at the age of sixteen.

### Young Persons Completing their Education.

A considerable number of young persons who will eventually come within the description of employed contributors are not entitled to become insured at the age of sixteen by reason of the fact that they are completing their education, and do not enter into contracts of service until a later age. To these young persons the Acts allow the full benefits, without requiring any additional contribution, provided it can be shown to the satisfaction of the approved societies which they join that since the attainment of the age of seventeen up to the date of their entry into insurance they have been at school or college, in unpaid indentured apprenticeships, or have otherwise spent their time in the completion of their education. We foresee some difficulty in determining whether or not a person is entitled to this consideration. The regulations issued by the Commissioners, as well as the section of the original Act, stipulate that the time must be spent "in a school or college," and although this phrase would apparently not exclude those who are not residents or boarders at such institutions, yet no ruling is given as to what amount of daily instruction is essential in order that the person may be regarded as spending his time "in the completion of his education"; it is also not apparent whether or not those who obtain instruction wholly in the evening would be excluded. It should, furthermore, be observed that the regulations distinctly lay down the rule that the whole of the time from the age of seventeen "up to the date of entry into insurance" must be spent in this way, and information is lacking as to what is the maximum period that will be allowed to elapse between the cessation of education and the commencement of ordinary employment. The point is of considerable importance, as it very frequently happens that a person leaves school before he has obtained a situation, and some time may elapse before employment is secured. This would appear to be a case in which the regulations should be interpreted according to the spirit and not according to the letter. However this may be, persons who are entitled to pay contributions and receive benefits at the original statutory rates should be careful to see that they receive the full benefits to which they are entitled.

### Persons Now Entering at Reduced Rates of Benefit.

There is, however, another class of persons who are not so fortunately situated in regard to their insurance. Many persons will become compulsorily insurable as years go by who have long ago attained the age of seventeen, but who have not previously had the right to claim the advantages of the National Insurance scheme.

These persons are no longer to be entitled to receive full benefits for the ordinary level contribution, but are to suffer a reduction of sickness benefit or in some way to make up the contributions to the extent that may be required. Such reduced sickness benefit will in no case be less than 5s. per week, and varies in the case of men from 9s. 6d. per week in respect of persons between the age of seventeen and eighteen at entry to 5s. per week for all men who are thirty or over when they first become insured. In the case of women the rate varies from 7s. per week for those between the age of seventeen and eighteen at entry to 5s. per week for all women aged twenty-one or over at entry.

The institution of a minimum sickness benefit of 5s. per week, while doubtless the product of admirable intentions, results in a certain amount of inequity as between the entrants at various ages. It will be readily appreciated that the general idea of the reduction in benefit to late entrants is to ensure that the contributions paid by and on behalf of the insured persons shall in themselves be adequate to provide the benefits received, and thus to avoid the necessity of creating book debts or reserve values.

Now it will be apparent, since the contribution is constant, that if the true equivalent sickness benefit be paid, such sickness benefit will continually decrease with an increase in the entry age. In actual fact, however, a minimum sickness benefit of 5s. a week is guaranteed under the Act of 1911, and it thus follows that at all ages above that at which 5s. per week sickness benefit is equivalent to the contribution paid in respect of it, each employed contributor will receive more benefit than he pays for, and all entrants at these ages would therefore in ordinary circumstances cause a loss to the society. This fact has been recognised by the Commissioners, who have arranged that the societies shall be credited with a "reserve value" equivalent to this loss.

### "Reserve Values."

The inequity of the arrangement will now become clear. These "reserve values" will be gradually liquidated, together with the "reserve values" set up in respect of those who entered within the first fifteen months, by deductions from the contributions of all employed contributors. The younger entrants among those who enter after October 12, 1913, will thus be called upon to assist not only in the liquidation of the reserves in respect of the original entrants, but in making good a loss that arises through granting too favourable terms to the older members of the same class as themselves. In this instance, therefore, as in others, the younger members have to pay in some degree for the older. The disturbance of equity was perhaps to an extent unavoidable, but its existence should be recognised, and we feel justified in saying that it was not generally realised that there would be any necessity for setting up further "reserve values" after the original period for entry at full rates had expired. It is also difficult to see that there will be any possibility of reaching finality in the liquidation of the reserves if further reserves are constantly being set up.

### How Full Rates of Benefit May be Secured.

We now come to a point which we particularly desire to emphasise, and that is that the reduction of benefit described above may be avoided by the employed contributor should he so desire.

Qualification for full benefits may be effected in either of two ways: (*a*) by paying to the Commissioners a lump sum varying according to the age at entry, or (*b*) by making up the weekly contribution actually paid at the employed rate as from the date of entry to the voluntary rate appropriate to the entry age. The lump sum that would be payable if the former course were adopted varies according to the sex of the employed contributor, and in the case of a woman it also differs with her status—that is, according as she is a spinster, a married woman, or a widow. In the case of men the amount to be paid increases from 9s. at the age of seventeen at entry to a maximum of £4 19s. for the ages of thirty-six to forty, and thereafter decreases to 6s. at the age of sixty-nine. The progression of the amounts is very similar for women, the maximum being £2 7s. for spinsters and widows of the ages of forty-four to forty-nine, and £2 10s. 6d. for married women who enter at the ages of thirty-six to forty-two.

It will be clear that if the reduced benefits payable to late entrants were the true equivalents to the contributions, the lump sums payable to provide full benefits without an increase in contribution should be exactly equal to the "reserve values" allocated to insured persons of similar ages at entry who entered within the days of grace. This is actually the case up to about the age of thirty at entry, but after that age the lump sums payable fall considerably short of the "reserve values." This fact demonstrates very clearly the inequity that we pointed out earlier in this article, since the difference at any age between the lump sum payable to secure full benefits and the reserve value that would have been credited if the insured person had entered within the days of grace represents very well the monetary advantage secured by the older members.

### The Second Alternative.

Should the insured person desire to take advantage of the second alternative—viz., that of making up the difference between the employed and voluntary rates—he may readily do so, but it is to be remembered that no part of the extra charge is to be borne by the employer.

With regard to the relative advantages of payment in a lump sum or payment of the difference in the weekly contributions, the latter would seem to be distinctly more disadvantageous to the older members. The voluntary rate of contribution is the full contribution necessary to provide the benefits without the assistance of any reserve value. If, therefore, the employed contributor makes up his contributions to the voluntary rate, it will not be necessary to credit the society with a "reserve value" in respect of his insurance. It has already been shown that a book debt must be created in respect of the older members if they suffer the reduced sickness benefit or pay a lump sum to qualify for full benefits, and the very fact, therefore, that such book debt is not required if the member adopts this second alternative is sufficient evidence that he is getting relatively less benefit for his contributions.

### Another Method of Qualifying.

It may be pointed out that another method of qualifying for full benefits is contained in a proviso to Sub-section (4) of Section (9) of the principal Act. It is there laid down that if a person such as we have been considering so desires he may consider himself as having entered into insurance at the age of seventeen, and to be in arrear for all contributions at the employed rate for the period elapsing between the age of seventeen and his actual age at entry. It will be clear that in certain circumstances, particularly if he is not much past the age of seventeen at entry, such a course would entitle him to a higher rate of benefit than he would otherwise receive, and if this is so he is given the opportunity of taking advantage of it.

It is not apparent in what way the new arrears regulations, with which we dealt last week, will affect this proviso, but the latter is still in force, and might well be adopted when such a course is to the insured person's monetary advantage.

